# The incremental treatment of ESRD: a low-protein diet combined with weekly hemodialysis may be beneficial for selected patients

**DOI:** 10.1186/1471-2369-15-172

**Published:** 2014-10-29

**Authors:** Stefania Caria, Adamasco Cupisti, Giovanna Sau, Piergiorgio Bolasco

**Affiliations:** Nephrology and Dialysis Unit, ASL 8, Cagliari, Italy; Department of Clinical and Experimental Medicine, University of Pisa, Pisa, Italy; Nephrology and Dialysis Unit, Brotzu Hospital, Cagliari, Italy

**Keywords:** Nutrition, Low protein diet, Hemodialysis, CKD, ESRD, Incremental dialysis

## Abstract

**Background:**

Infrequent dialysis, namely once-a-week session combined with very low-protein, low-phosphorus diet supplemented with ketoacids was reported as a useful treatment schedule for ESRD patients with markedly reduced residual renal function but preserved urine output. This study reports our findings from the application of a weekly dialysis schedule plus less severe protein restriction (standard low-protein low-phosphorus diet) in stage 5 CKD patients with consistent dietary discipline.

**Methods:**

This is a multicenter, prospective controlled study, including 68 incident CKD patients followed in a pre-dialysis clinic with Glomerular Filtration Rate 5 to 10 ml/min/1.73/ m^2^ who became unstable on the only medical treatment. They were offered to begin a Combined Diet Dialysis Program (CDDP) or a standard thrice-a-week hemodialysis (THD): 38 patients joined the CDDP, whereas 30 patients chose THD. Patients were studied at baseline, 6 and 12 months; hospitalization and survival rate were followed-up for 24 months.

**Results:**

Volume output and residual renal function were maintained in the CDDP Group while those features dropped quickly in THD Group. Throughout the study, CDDP patients had a lower erythropoietin resistance index, lower β2 microglobulin levels and lower need for cinacalcet of phosphate binders than THD, and stable parameters of nutritional status. At 24 month follow-up, 39.4% of patients were still on CDDP; survival rates were 94.7% and 86.8% for CDDP and THD patients, respectively, but hospitalization rate was much higher in THD than in CDDP patients. The cost per patient per year resulted significantly lower in CDDP than in THD Group.

**Conclusions:**

This study shows that a CDDP served to protect the residual renal function, to maintain urine volume output and to preserve a good nutritional status. CDDP also blunted the rapid β2 microglobulin increase and resulted in better control of anemia and calcium-phosphate abnormalities. CDDP was also associated with a lower hospitalization rate and reduced need of erythropoietin, as well as of drugs used for treatment of calcium-phosphate abnormalities, thus leading to a significant cost-saving. We concluded that in selected ESRD patients with preserved urine output attitude to protein restriction, CDDP may be a beneficial choice for an incremental hemodialysis program.

## Background

The Epidemiology of chronic kidney disease (CKD) is on an upward trend worldwide. Since the 1990s incidence has risen by approximately 40% per decade, with prevalence rates in the general adult population up to 13% in the USA and ranging 10.2-11.6% in Europe [1–4]. These figures indicate a CKD epidemic leading to an increased number of patients requiring dialysis and a heavy burden on public health resources.

Nutritional and pharmacological therapy constitute the basis for the prevention and treatment of signs and symptoms of CKD, and they serve to delay the commencement of dialysis. The appropriate time to start dialysis is still a matter of debate. In particular, dialysis is not superior that conservative treatment especially in the comorbid-elderly population. It is known that a standard thrice--weekly hemodialysis schedule invariably leads to rapid loss of the residual renal function, to psychological and socials drawbacks, and to high costs. Real incremental dialysis programs are implemented in peritoneal dialysis but not in the hemodialysis setting where only a twice -weekly schedule may be proposed prior to maintenance hemodialysis.

During the 1980’s and 1990’s, an Integrated Diet Dialysis Program (IDDP) was proposed as a therapeutic option for selected patients with markedly reduced residual renal function and it consisted of weekly hemodialysis session (HD) combined with nutritional therapy, namely a very low-protein diet supplemented with ketoacids [5–8]. Malnutrition risk and low compliance to the severe dietary restrictions represented major concerns that prevented a widespread application of this schedule. In the present study we propose a Combined Diet Dialysis Program (CDDP) which includes a less severe low protein (0.6 g/Kg/d) diet combined with once-weekly hemodialysis. The goal is to prolong a conservative approach and thus reduce the need for hemodialysis, thereby limiting the risk of malnutrition and of poor dietary adherence. This study aimed to assess the safety, benefits and drawbacks of the CDDP approach including nutritional status, residual renal function, morbidity, mortality and costs.

## Methods

This is a multicenter, non-randomized, prospective controlled study, including stage V CKD patients followed in a pre-dialysis clinic who were not suitable for a peritoneal dialysis program.

Patients with GFR 5 to 10 ml/min who were approaching hemodialysis treatment and who had vascular access were included in the study. Global criteria of starting dialysis and then for entering the study were fluid retention, inadequate control of BUN or severe secondary hyperparathyroidism or hyperphosphatemia, reduced compliance to dietary treatment.

Patients with pericarditis, congestive heart failure, severe fluid retention, overt protein-energy wasting, hyperkalemia or severe metabolic acidosis with acute reduction of residual renal function or concurrent diseases were excluded, as well patients with unreliable discipline with regards to dietary restrictions were treated at once with maintenance hemodialysis and then excluded from the study. During the pre-dialysis phase, patients were followed in a tertiary care CKD clinic every two-three months (stage 4) or on a monthly basis (stage 5). Compliance to dietary and pharmacological treatment was acceptable, as assessed by urinary urea excretion and by dietary recall and interview.

Sixty-eight incident patients were recruited and all the patients were provided a functioning native artero-venous fistula or graft.

Underlying kidney diseases included mainly hypertension/vascular nephropathy, ADPKD. The patients were offered the choice to commence a weekly hemodialysis schedule plus low-protein diet on the non-dialysis day (namely, CDDP), or to begin a standard thrice- weekly hemodialysis (THD) schedule and free-choice diet. All the patients were informed and directly involved in the decision making process. Randomization was not applied since any dietary regimen requires adherence and motivation.

According to their own choice, 38 patients entered in the CDDP Group, whereas the remaining 30 patients formed the THD control group. The clinical and biochemical features of the two groups at baseline are reported in Table 1. At baseline, the prevalence of cardiovascular disease history was similar in CDDP (28.9%) and in THD (30%) group. Six type 2 diabetics were included in CDDP group and 13 in THD group.

**Table 1:** **Baseline characteristics of the studied groups**

	CDDP group (n = 38)	THD group (n = 30)	p
Male/females	25/13	19/11	
Age, years	64.5 ± 13.2	65.2 ± 11	*0.82*
Body weight (kg)	65.5 ± 15.1	66.2 ± 11.9	*0.73*
BMI (kg/m^2^)	23.7 ± 4.0	25.6 ± 4.13	*0.03*
Urine volume output (mL/24 h)	1983 ± 651	1472.6 ± 433	*<0.001*
GFR (mL/min × 1.73 mq b.s)	7.8 ± 1.9	9.2 ± 4.2	*<0.01*
EPO (IU/kg/week)	104 ± 108	184 ± 84	*<0.001*
CRP <5 mg/dl, %	89	66.6	*<0.01*
iPTH, >300 ρg/mL, %	31.5	50	*<0.01*
Charlson comorbidity index score	5.5 ± 2.5	3.8 ± 2.5	*0.004*
Charlson comorbidity index score >4, %	62.5	33.3	*<0.01*

All the patients were studied at baseline, and after 6 and 12 months. The survival rate and hospitalization rate were followed up to 24 months.

Blood samples were collected from the arterial line before the start of the first dialysis of the week; 24h urines were collected during the day before the HD session. GFR was measured as the average of creatinine clearance and urea clearance, and expressed as ml/min*1.73 m^2^ body surface area [9]; eGFR was also estimated by MDRD formula [10]. Protein catabolic rate (PCR) was used as an indicator of dietary protein intake and calculated by the urea appearance method according to Maroni’s formula [11]. Maroni’s formula was used throughout the course of the CDDP and only at baseline time in patients in the THD Group. Within the THD Group, for patients with the significant loss of renal function and urine output volume we included the method of urea kinetics model for estimation of nPCR. Dialytic adequacy was expressed using eKt/V according to Daugirdas formula et al [12]. Body mass index was calculated by the body weight at the end of the dialysis session. The bioelectrical parameters, were measured with a single-frequency impedance analyzer (BIA 101 – Akern - Florence), 30’ after the end of the hemodialysis session.

Erythropoietin Resistance Index (ERI) was calculated as weekly EPO dosage (units)/body weight (Kg)/Hb (g/dl) [13].

Charlson’s comorbidity index (CCI) score, was calculated to evaluate comorbidity conditions [14].

Creatinine production, as a surrogate of muscle mass, was calculated by the sum of 24h urine creatinine excretion and metabolized creatinine. Metabolized creatinine was calculated from the product of serum creatinine and extrarenal Creatinine clearance (estimated as 0.038 L/kg BW/d) [7].

All the patients on CDDP underwent nutritional counseling by a renal dietician and were prescribed a low protein (0,6 g/kg b.w./d), low phosphorus (600–700 mg/d), low sodium diet with an energy intake of 30–35 kcal/kg b.w./d. Proteins were mostly from animal sources (0.4 g/Kg/b.w.), to cover the requirement of essential amino acids; no dairy or processed foods are included to the aim of limiting the sodium and phosphorus intake; [15] protein-free products are included to supply energy with negligible load of phosphorus, sodium, potassium and nitrogen [16]. The 60–62% of energy intake derived from carbohydrates (mostly represented by protein-free products), 30–32% from lipids and 8-10% from proteins. As average, the daily dietary plan consisted of 5–7 servings of protein free products, namely 2–3 serving for breakfast and snacks and 2 servings for both lunch and dinner; the patients were usually advised that they can add other servings, when required to satisfy the energy requirement. The servings of animal protein sources (meat, fish, egg white) were calculated for each patient in order to guarantee a daily total amount of 0.4 g protein per kg b.w. Two servings of vegetables and two of fruits were provided with specific suggestions for patients with high potassium levels as well as boiling was suggested to reduce to mineral content (potassium, sodium, calcium, and phosphorus) of food. Finally, 5–6 servings of fats (olive oil as first choice) were suggested.

This diet was prescribed for 6 days a week, while no dietary restrictions occurred on the dialysis day.

Patients in the THD control group followed an unrestricted protein diet, but they were given dietary counseling focused on preventing excess sodium, potassium, phosphorus and fluid intakes.

All the patients were prescribed 4-hour hemodialysis sessions with highly biocompatible synthetic membranes.

The study protocol was approved by the Cagliari Hospital Ethics Committee, and all the patients gave their written informed consent for participation in the study.

### Statistical analysis

Descriptive statistics is given as mean ± standard deviation. Normality tests were performed on all continuous variables measured throughout the study. The log-rank test was used for hypothesis testing. Time repeated measurements were analyzed using linear mixed models including treatment, time, and treatment by time interaction for all measured variables using ANOVA. Inter-group drug use was compared by means of the binomial test with Bonferroni’s correction for multiple comparisons.

Differences were considered as statistically significant when p <0.05.

## Results

Patients who entered the CDDP showed several differences with respect to patients who commenced THD (Table 1). Despite a lower residual renal function (RRF), CDDP patients showed lower circulating levels of phosphate, BUN, PTH and higher albumin levels with respect to THD patients. 34 out of the 38 patients were still on CDDP at 12 months: 2 patients were shifted to a thrice- weekly dialysis schedule at 8 and 10 months, while 2 patients partially recovered their renal function and remained clinically stable with the low-protein diet and no need for dialysis.

**Figure 1:**
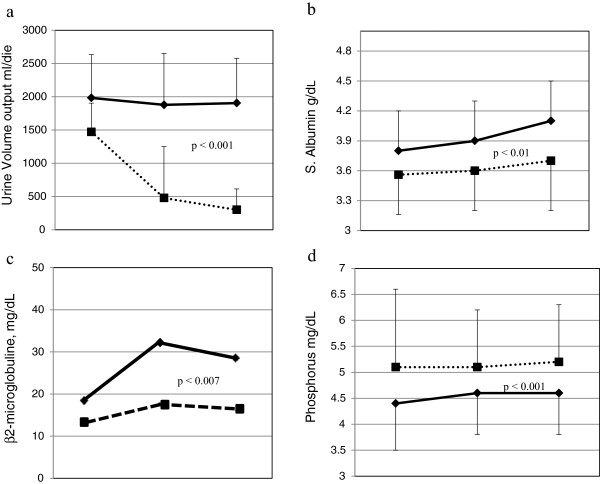
**Changes in urine volume (a), serum albumin (b), β2-microglobulin (c) and serum phosphorus (d) in CDDP Group (dotted Line) and THD Group (full line), at baseline, 6 and 12 months of follow-up.**

During the study period, GFR was preserved in CDDP patients in comparison to THD group (Table 2). GFR loss progression was very low in the former (-0.13 ml/min/month) while it was faster in the latter (-1.53 ml/min/month). Similarly, in the CDDP group an effective urine volume output was maintained whereas it dramatically dropped in the THD patients (Figure 1a). As a consequence, the interdialytic weight gain in CDDP patients was limited to (800 ± 300 g) per week. The dosage of frusemide increased from 150 ± 154 to 273 ± 201 mg/day in the CDDP group, and from 332 ± 171 to 409 ± 205 mg/day (p =0.003) in the THD group. The increase of β2-microglobulin circulating levels was much lower in CDDP than in THD patients (+11.2% vs +34%, respectively) (Figure 1c).

**Table 2:** **Outcome of nutritional and functional parameters in both groups in 12 months**

	CDDP group			THD group			
	Baseline (n = 38)	6 months (n = 38)	12 months (n = 34)	Baseline (n = 30)	6 months (n = 30)	12 months (n = 29)	***P***
*Dry weight (kg)*	*65 ± 15.0*	*63.9 ± 14.4*	*63.4 ± 14.7*	*66.2 ± 11.9*	*64.9 ± 11.5*	*65.5 ± 12.3*	*0.07*
*BMI (kg/m* ^*2*^ *)*	*23.7 ± 4.0*	*23.4 ± 3.7*	*23.0 ± 3.9*	*25.6 ± 4.13*	*25.1 ± 4.07*	*25.4 ± 4.2*	*0.07*
*Total body water (L)*	*44.4 ± 10.5*	*41 ± 9.6*	*43 ± 11*	*35.4 ± 6*	*33.5 ± 5.8* ^*a*^	*33.8 ± 5.9* ^*a*^	*0.02*
*Extracellular water (L)*	*20 ± 4.5*	*18.6 ± 4.3*	*19.7 ± 4*	*17.5 ± 3.2*	*16 ± 2.8* ^*b*^	*15.7 ± 2.4* ^*b*^	*0.04*
*Fat mass (kg)*	*11 ± 12.3*	*13 ± 11.8*	*11.4 ± 11*	*20 ± 9*	*21.4 ± 8.5*	*22.6 ± 9.4*	*0.03*
*Body cell mass (kg)*	*31 ± 9.6*	*28 ± 8.9*	*29.7 ± 10.3*	*22.5 ± 6.2*	*22.7 ± 5*	*22.7 ± 5.8*	*0.02*
*Phase angle, (°)*	*6.2 ± 1.3*	*6.2 ± 1.3*	*6.1 ± 1.4*	*5.0 ± 1.2*	*6 ± 0.9*	*5.8 ± 1.2*	*0.06*
*SBP (mmHg)*	*139 ± 18*	*132 ± 20*	*128 ± 15* ^*b*^	*136.8 ± 17.2*	*138.5 ± 18.5*	*139 ± 20.8*	*0.21*
*DBP (mmHg)*	*80 ± 12*	*73 ± 10*	*74 ± 9* ^*c*^	*71.6 ± 11.4*	*71.6 ± 10*	*72.9 ± 12.3*	*0.08*
*Serum creatinine (mg/dL)*	*6.4 ± 1.9*	*7.0 ± 2.5*	*7.8 ± 3.0* ^*b*^	*5.9 ± 1.8*	*7.8 ± 2.2*	*8.2 ± 2.4* ^*b*^	*0.05*
*B.U.N. (mg/dL)*	*68 ± 18*	*68 ± 16*	*70 ± 17*	*84.4 ± 18.5*	*77 ± 15.5*	*77 ± 22.9*	*0.003*
*GFR (mL/min)*	*7.8 ± 1.9*	*6.7 ± 2.3*	*6.3 ± 2.1* ^*b*^	*9.2 ± 4.2*	*-*	*-*	*-*
*eGFR (mL/min)*	*9.4 ± 2.9*	*8.4 ± 3.5* ^*a*^	*8.0 ± 3.4* ^*b*^	*10.3 ± 3.9*	*-*	*-*	*-*
*Total protein (g/dL)*	*6.7 ± 0.5*	*6.7 ± 0.5*	*6.8 ± 0.4*	*6.35 ± 0.52*	*6.4 ± 0.5*	*6.5 ± 0.43*	*0.004*
*Albumin (g/dL)*	*3.8 ± 0.4*	*3.9 ± 0.4*	*4.1 ± 0.4* ^*b*^	*3.6 ± 0.40*	*3.6 ± 0.37*	*3.7 ± 0.48*	*0.01*
*Transferrine (mg/dL)*	*219 ± 53*	*203 ± 72*	*222 ± 53*	*264 ± 94*	*249 ± 43*	*251 ± 61*	*0.001*
*C3 (mg/dL)*	*90 ± 20*	*91 ± 23*	*94 ± 23*	*99.6 ± 39.6*	*96 ± 35.7*	*95.9 ± 32*	*0.48*
*C4 (mg/dL)*	*24 ± 7*	*24 ± 8*	*26 ± 7*	*33 ± 28*	*29 ± 18*	*29 ± 30*	*0.26*
*Total IgG (mg/dL)*	*1599 ± 497*	*1590 ± 402*	*1543 ± 373*	*1440 ± 461*	*1482 ± 487*	*1409 ± 413*	*0.21*
*Lynphocytes/mm* ^*3*^	*1539 ± 651*	*1390 ± 831*	*1581 ± 537*	*1452 ± 811*	*1458 ± 622*	*1445 ± 580*	*0.38*

Legend: changes versus baseline: ^a^p < 0.05; ^b^p < 0.01; ^c^< 0.03; **P:** ANOVA significance for treatment by time interaction.

No difference in arterial blood pressure values emerged between the two groups.

The main nutritional indicators, i.e. total protein, albumin and transferrin improved significantly in the CDDP group (Figure 1b, Table 2). Estimated dietary protein intake remained stable in the CDDP groups throughout the study (from 0.58 ± 0.2 to 0.57 ± 0.11 g/kg/day). This was also the case for THD patients (from 1.03 ± 0.1 to 1.04 ± 0.3 g/kg/day). No significative changes in body weight or Body Mass Index (BMI) were recorded in the two groups (Table 2).

A significant reduction in the ESAs requirement was observed in the CDDP group along with markedly lower ERI values (Table 3).

**Table 3:** **Biochemistry parameters in CDDP and THD groups at 6 and 12 months**

	CDDP group			THD group			
	Baseline (n = 38)	6 months (n = 38)	12 months (n = 34)	Baseline (n = 30)	6 months (n = 30)	12 months (n = 29)	P
*Triglycerides (mg/dL)*	109 ± 36	134 ± 92	126 ± 50	131 ± 72	152 ± 67	139 ± 52	*0.03*
*Total Cholesterol (mg/dL)*	164 ± 36	168 ± 36	164 ± 35	165 ± 41	168.5 ± 34	170 ± 40	*0.51*
*HDL Cholesterol (mg/dL)*	44 ± 14	47 ± 13	48 ± 20	44 ± 12	45 ± 13	44 ± 12	*0.58*
*LDL Cholesterol (mg/dL)*	101 ± 29	93 ± 34	93 ± 31	95 ± 32	93 ± 29	97.8 ± 34	*0.95*
*Cholinesterases (UI/mL)*	5474 ± 1396	6073 ± 1474	586 ± 1646	4968 ± 1488	5511 ± 1604	4673 ± 1450^b^	*0.27*
*Uric acid (mg/dL)*	7.3 ± 2.3	7.5 ± 2.4	7.1 ± 1.9	6.4 ± 1.8^b^	7.3 ± 1.2	7.2 ± 1.3^b^	*0.60*
*Sodium (mmol/L)*	139 ± 2.6	137 ± 3.6	137 ± 4.0	138 ± 2.7	138 ± 2.3	134 ± 3.3	*0.58*
*Potassium (mmol/L)*	4.4 ± 0.6	4.3 ± 0.6	4.3 ± 0.7	4.3 ± 0,67	4.7 ± 0.7	4.8 ± 0.9^b^	*0.07*
*Calcium (mg/dL)*	9.2 ± 0.5	9.0 ± 0.5	9.1 ± 0.6	9.1 ± 0.6	9.1 ± 0.7	9.0 ± 0.5	*0.51*
*Phosphatemia (mg/dL)*	4.4 ± 0.9	4.6 ± 0.8	4.6 ± 0.8	5.1 ± 1.5	5.6 ± 1.1	5.2 ± 1.1	*0.001*
*Bicarbonate (mEq/L)*	22.0 ± 3.1	23.1 ± 3.3	23.2 ± 3.2	21.8 ± 4.2	21.9 ± 3.2	21.6 ± 2.7	*0.17*
*β2-microglobulin (mg/dL)*	14.2 ± 3.9	16.8 ± 5.7	16.0 ± 5.1	18.4 ± 11.6	31.0 ± 16.0	28.0 ± 11.4^b^	*0.007*
*Hb (g/dl)*	10.8 ± 0.1	11.5 ± 0.95	11.5 ± 0.97^a^	10.5 ± 1.4	11.3 ± 0.9	11.2 ± 0.95^a^	*0.31*
EPO (IU/kg/week)	104 ± 108	69 ± 59	60 ± 74^b^	184 ± 84	172 ± 138	204 ± 252	*0.002*
ERI (IU/kg/week)/Hb	10 ± 11	6 ± 5	5 ± 7^b^	19 ± 10	15 ± 13	19 ± 23	*<0.001*
*CRP (% patients) <5.0*	89	81.5	79	66.6	83.3	76.6	*<0.05*
*CRP (mg/dL)*	3.3 ± 3.9	3.5 ± 3.3	3.2 ± 3.6	8.6 ± 9.7	4.9 ± 6.5	5.8 ± 5.8	*<0.01*
*iPTH (ρg/mL) <150 (%)*	39.5	52.6	52	36.7	43.3	34.5	*0.09*
*iPTH (ρg/mL) 150–300 (%)*	29	29	30	13.3	20	27.6	*0.26*
*iPTH (ρg/mL) >300 (%)*	31.5^a^	18.4	18^a^	50	36.7	37.9	*0.02*

Changes versus baseline: ^a^p < 0.01; ^b^p < 0.03; **P:** ANOVA significance for treatment by time interaction.

Calcium phosphate metabolism parameters were stable and better controlled in the CDDP (Table 3, Figure 1d) group despite a significantly lower use of non-calcium containing phosphate-binders and cinacalcet and among all the drugs, only allopurinol use was reduced during THD (Table 4).

**Table 4:** **Pharmacologic treatments in CDDP and THD groups**

	CDDP Group		THD group		P
% of patients	Baseline	12 months	Baseline	12 months	
*ACEi*	34	33	50	48.3	*n.s*
*ARBs*	24	26	30	21	*n.s*
*Calcium channel blockers*	28.9	27.3	30	17	*n.s*
*Calcium carbonate*	59	60	76.6	72.4	*n.s.*
*Sevelamer or Lanthanum carbonate*	0	12^a^	0	41^c^	*<0.03*
*Paricalcitol*	0	12^b^	0	17.2^b^	*n.s.*
*Cinacalcet*	0	6^b^	0	24^c^	*<0.04*
*Calcitriol*	45	42	46.7	45	*n.s.*
*Kayexalate*	21	21	20	38	*n.s.*
*Allopurinol*	48.6	54	33.3	13.8^a^	*0.01*
*Statins*	37	33	26.7	24	*n.s*
*Polyunsaturated fats*	13	9	10	17	*n.s.*

Legend: Changes versus baseline: ^a^p<0.05; ^b^p<0.01; ^c^<0.03; **P:** ANOVA significance for treatment by time interaction.

Calcimimetics drugs are not delivered by Italian NHS during the pre-dialysis phase.

So their use began after commencing dialysis (even once-a-week): patients on THD showed a secondary hyperparathyroidism more severe than patients on CDDP , consequently the need of calcimimetics was higher in the former than in the latter.

No significant reduction in creatinine production was observed after 12 months of CDDP (16.8 ± 4.3 vs. 15.9 ± 3.9 mg/Kg/d).

### Hospitalization

Throughout the 24 month follow-up period, 3 CDDP patients were admitted to hospital for a total of 11 days (3.7 ± 1.5 days/patient) due to atrial fibrillation, acute bronchitis and acute cholecystitis. Instead 24 hospital admission were recorded in 15 patients of THD groups for a total of 147 days (6.1 ± 6.3 days/patient). Causes for admission were: set up of new vascular access (7), artero-venous fistula stenosis angioplasty (1), infection of central vein catheter (2), acute pulmonary edema (1), surgery for biological prosthetic valve (1), myocardial infarction (1), valvular and coronaropathy angioplasty (2), congestive heart failure (1), atrial fibrillation (2), hyperpyrexia (1), hypertensive crisis (1), hypoglycemic coma (1), abscess in thigh hematoma (1) and obstructive jaundice secondary to gallstones (2).

### Survival

**Figure 2:**
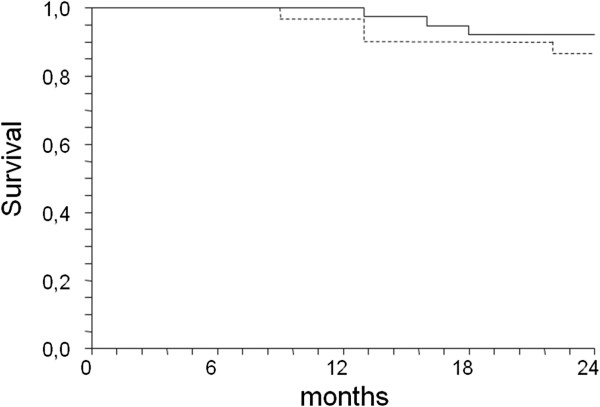
**Cumulative survival in CDDP group (Dotted line) and in THD Group (Full line).**

At the 24-month follow-up, no significant difference was detected between the CDDP and THD group survival rates: 94.7% and 86.8% respectively (Figure 2).

Death in 3 CDDP patients was caused by cardiac events: 1 during the 13th, 1 during the 16th and 1 during the 18th month of CDDP. Four deaths occurred in the THD group: 1 in the 9th, 2 in the 13th and 1 in the 22nd month following start of dialysis. Causes included myocardial infarction and acute heart failure, sepsis and stroke.

### CDDP global outcome

At 12 months 34 out of the 38 patients (89.5%) were on CDDP. At 24 months, 15 patients were still on CDDP (39.4%) since 1 patient received a kidney transplant, 3 patients had fatal myocardial infarction, whereas 17 patients have progressed to an incremental dialysis program (namely twice to thrice-weekly dialysis and free-protein diet) and 2 patients partially recovered their renal function. The main reasons for dropping out were loss of dietary adherence and/or reduction of residual renal function.

When compared to THD group, CDDP patients registered a lower number of hospitalizations and a lower need for expensive drugs. These findings, coupled with the 66% reduction in number of dialysis sessions (52 versus 156 sessions in THD Group in a year) allows to estimated that the cost of a CDDP patient is more than 60% less than that of a THD patient.

## Discussion

This study shows that in selected ESRD patients, a CDDP is able to protect the RRF, to maintain urine volume output, to preserve a good nutritional status, to blunt the rapid β2 microglobulin increase, and to allow better control of anemia and calcium-phosphate abnormalities. CDDP is also associated with a lower rate of hospitalization , reduced need of EPO and drugs used for the CKD-MBD treatment, thus leading to a significant cost-saving. CCDP may be considered as the first step of an incremental approach to hemodialysis treatment of ESRD.

Patients who entered the CDDP showed a better control of phosphatemia, BUN, PTH levels and higher albumin levels despite a lower RRF. It suggests that patients who had maintained consistent adherence to dietary treatment chose the CDDP whereas THD was chosen by patients with poor compliance to dietary prescriptions. In fact, dietary discipline is the first pre-requisite for a safe and successful CDDP.

In the CDDP group an effective urine volume output was maintained whereas it dramatically dropped in the THD patients. As a consequence, the interdialytic weight gain in CDDP patients was quite small, thus allowing low intradialytic ultrafiltration volumes. In turn, this may contribute to further preservation of the residual renal function and to limit cardiovascular damage. When compared to THD, CDDP was not associated with greater mortality risk (Figure 2) and hospitalization rate was much lower.

One of the main goals of the CKD patients care is to preserve RRF as long as possible because it is of very favorable prognostic value. The majority of patients succeeded in sticking to CDDP for at least one year ensuring a good nutritional status and maintaining GFR (-1.56 ml/min/year); in stage 4–5 CKD Levin et al. reported a GFR loss of 2.6 ml/min/year [17].

Three major studies investigated once a-weekly dialysis coupled with low protein regimens in ESRD patients [5–8]. Mitch et al. reported that combined diet-dialysis treatment resulted in a decrease in urea production with a positive nitrogen balance during interdialytic interval [5]. Patients studied by Giovannetti et al. showed no uremic symptoms even after years of IDDP despite an extremely low RRF, thus indicating that the nutritional therapy is a major factor preventing the onset of uremic symptoms [6]. The studies carried out by Locatelli et al. concluded that IDDP may be very important from a psychological and economic point of view, but concerns arose about compliance and long-term nutritional and depurative adequacy [7]. After a 4-year period they found a reduction of creatinine generation rate, possibly suggesting a reduction in muscle mass.

This was not confirmed in our patients where the CDDP differs from the similar strategies proposed in the 1980’s and 1990’s [6, 7]; the treatment is distinguished by less severe residual renal function from among patients, less severe protein restriction, and by closer nutritional monitoring. In addition, hemodialysis sessions were performed with high quality water and highly bio-compatible membranes [18–21].

Retrospective and prospective studies failed to demonstrate any benefits, in terms of survival, for an early onset of dialysis. In 2010 the first randomized, controlled trial of early versus late initiation of dialysis reported that early access to dialysis (eGFR > 7 ml/min/1.73 m^2^) provided no statistically significant benefits in terms of patient survival [22].

The role played by RRF in the clearance of medium-sized molecules such as β2-microglobulin is well-known (Figure 1c). Accordingly, patients with a significant RRF manifest lower levels of β2-microglobulin. On commencing CDDP, maintenance of effective urine output volume, a better hyperparathyroidism control, and a good nutritional status may have produced a positive effect on erythropoiesis leading to better anemia correction with reduction of ERI [23–25]. Our findings are in keeping with those of Di Iorio et al. who demonstrated that a poorer response to EPO was related to secondary hyperparathyroidism [23]. At baseline, in 50% of THD patients PTH was >300 pg/ml with significantly higher serum phosphate levels and a significantly lower use of calcimimetic drugs compared to CDDP Group (Tables 3 and 4). During the study, CDDP patients had no need for additional dialysis sessions. In turn, an easy volumes balance with quite low interdialytic weight gains, and prevention of high ultrafiltration rates and of intradialytic hypotension, contribute to the protection of RRF during CDDP. Moreover, Fouque et al. showed that dietary treatment produced a positive effect in delaying the need of dialysis [26].

It is noteworthy that the hospitalization rate in CDDP was much lower than in THD Group. In the latter, the causes of admission were mainly vascular access complications and infection. In addition the overall 2 year-survival rate of CDDP was similar to that of THD patients. In fact, data from literature show that a conservative approach may be not inferior to dialysis in elderly ESRD patients, in terms of survival and/or quality of life [27–29].

Lastly, the CDDP treatment resulted in an approximate 50% savings on the cost of drugs and dialysis resources, including a lower number of dialysis hours/nursing and medical staff ( 66% less compared to thrice-weekly hemodialysis) and comorbid incidence in terms of days of hospitalization, as well as of indirect costs such as transport of patients to and from the dialysis unit. When compared to THD, the CDDP group showed a lower hospitalization rate, lower need for expensive drugs, and by definition a 66% lowering of number of dialysis sessions and related costs. CDDP patients may have the additional cost of the protein-free products. As a whole, CDDP seems to be a safe and effective exit strategy for lowering the cost of ESRD population, even if it can occur for a limited period of time and in selected ESRD patients.

Another advantage of CDDP is an improvement in patient’s acceptance and adaptation to renal replacement therapy. The patient feels less sick and less machine-dependent, resulting in an increased copying capacity (or psychological adaptation). Patients moreover tended to adapt better to low protein diets thanks to a more varied diet, as well as to address concerns about “dialysis-day”, which was also the day they were allowed to eat a “normal” diet.

The study has not had the opportunity to randomize and this may be seen as a limitation, but randomization was not feasible because one could not force patients to choose one method rather than the other. Patients were followed in pre-dialysis care: a multidisciplinary clinic (doctors, nurses, dietitians and psychologists) was able to provide the necessary education and choice of treatment modalities. This study aimed to reproduce the real world clinical practice where patient’s adherence is a crucial issue for the success of any treatment, especially if dietary in nature, where patients play an active role and must be involved in the decision-making process. Patients who entered the CDDP showed a lower residual renal function but better control of phosphatemia, BUN, PTH levels and higher albumin levels. This finding suggests that CDDP is suitable for patients who still have a positive inclination towards dietary manipulations. Moreover, together with a preserved urine output volume, good compliance to dietary prescriptions is mandatory and a pre-requisite for the success and safety of CDDP. Now it could be necessary to begin considering a tailored dialytic treatment and to evaluate the greater power of RRF. The incremental choice is considered a good option in peritoneal patients since the 2000’s [30, 31] thanks to RRF and it again underestimates for twice-weekly HD patients [32, 33]. Energy-adequate low-protein regimens have a fundamental role in controlling and maintaining a good metabolic status and in reducing GFR loss [26, 34].

## Conclusions

This study shows that in selected ESRD patients, a CDDP is able to protect the RRF, to maintain urine volume output, to preserve a good nutritional status , to blunt the rapid β2 microglobulin increase, and to allow better control of anemia and calcium-phosphate abnormalities. CDDP is also associated with a lower rate of hospitalization and reduced need of EPO and drugs used for the CKD-MBD treatment, thus leading to a significant cost-saving. As a whole, our findings suggest that CDDP could be a beneficial choice for selected collaborative patients who would otherwise be referred to thrice-weekly hemodialysis. CCDP should be considered as the first step of an incremental approach to hemodialysis treatment in motivated and selected ESRD patients, or anywhere dialysis facilities and resources are lacking.

## Abbreviations

ADPK: Autosominal dominant polycystic kidney disease
CDDP: Combined diet dialysis program
THD: Thrice-weekly dialysis
IDDP: Integrated diet dialysis program
RRF: Residual renal function
CKD: Chronic kidney disease
ESRD: End stage renal disease
GFR: Glomerular filtration rate
eGFR: Estimated glomerular filtration rate
CKD-MBD: Chronic kidney disease- mineral bone disorders
HD: Hemodialysis
ERI: Erythropoietin resistance index.
